# Supplementary material for: Genetic and Developmental Divergence in the Neural Crest Program between Cichlid Fish Species
Source: Mol Biol Evol. 2024 Oct 16;41(11):msae217. doi: 10.1093/molbev/msae217 (PMC11558072; doi:10.1093/molbev/msae217)
Supplement: msae217_Supplementary_Data [file msae217_supplementary_data.zip › Supplementary Figure S1.docx]

**
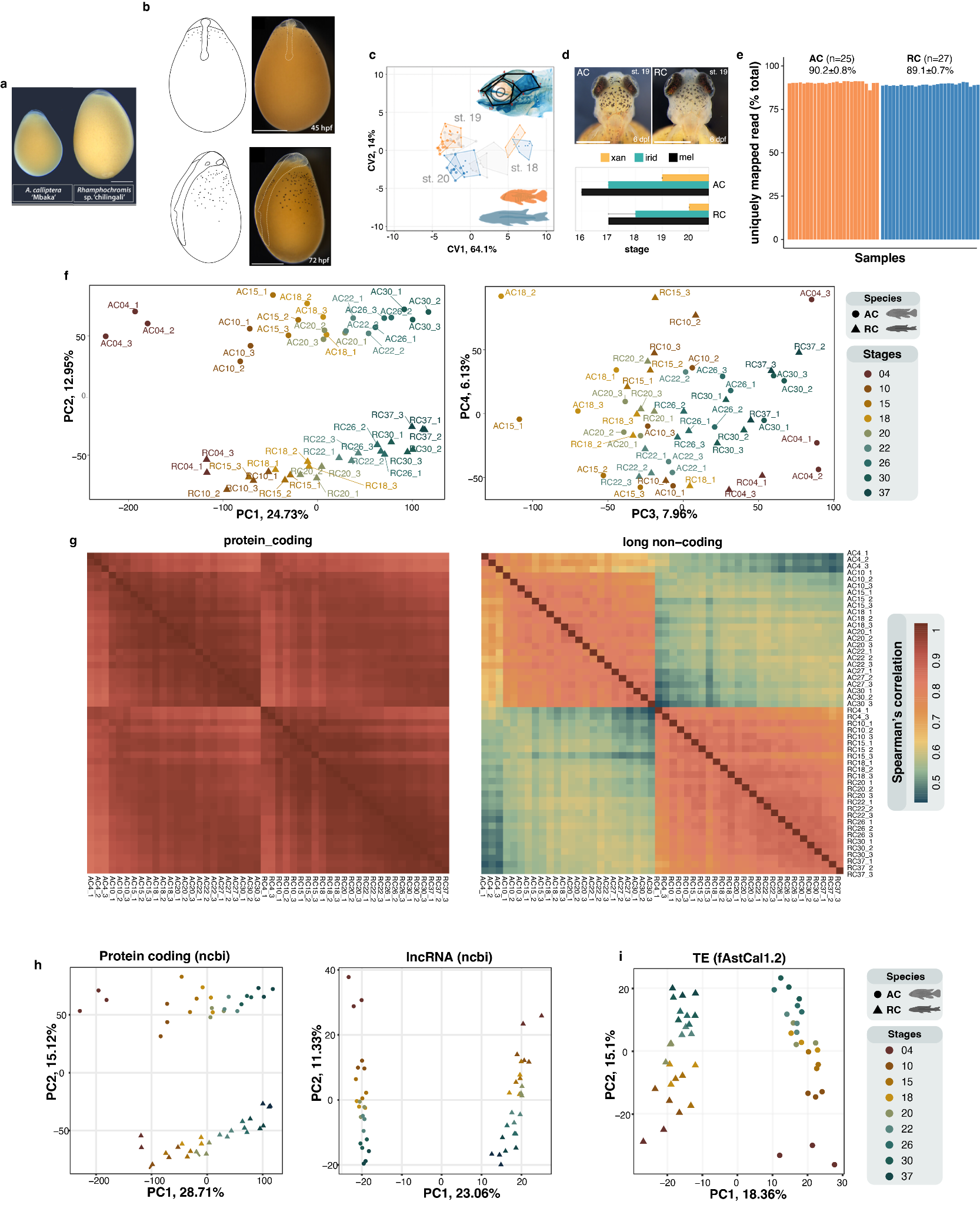
**

**Supplementary Figure S1. Comparative characterization of cichlid transcriptomic landscapes during somitogenesis and neural crest development.** **a)** Images of fertilized eggs for AC (left) and RC (right). **b)** Example of embryo dissected from the yolk. **c-d**) Interspecific divergence in shapes of the craniofacial skeleton (depicted in a common morphospace in **c**), and timing of pigment cell appearance (**d**) during post-hatching development (stages 16-20) indicates that phenotypic variation in these NC-derived traits is specified prior to their overt formation (Marconi et al. 2023)**. e)** Mapping rates for all sequenced samples. Number of biological replicates per group and mean ± standard deviation of mapping rates are given above the graph. **f)** PCA plots showing PC1-PC2 (left) and PC3-PC4 with labeled samples (right) as part of the whole transcriptome analysis (see Fig. 1e). **g)** Heatmaps showing pairwise Spearman’s correlation scores of gene expression values for protein coding (23,664 transcribed genes with ≥5 normalized count in any one sample) and non-coding transcripts (1,664 transcribed genes with ≥5 normalized count in any one sample). **h-i**) PCA plots of gene expression values (DESeq2 gene count normalization) for protein coding and lncRNA (**h**), and for transcribed TE transcripts (1,609 TE transcripts) (**i**).
