# Supplementary material for: Genetic and Developmental Divergence in the Neural Crest Program between Cichlid Fish Species
Source: Mol Biol Evol. 2024 Oct 16;41(11):msae217. doi: 10.1093/molbev/msae217 (PMC11558072; doi:10.1093/molbev/msae217)
Supplement: msae217_Supplementary_Data [file msae217_supplementary_data.zip › Supplementary Figure S2.docx]

**
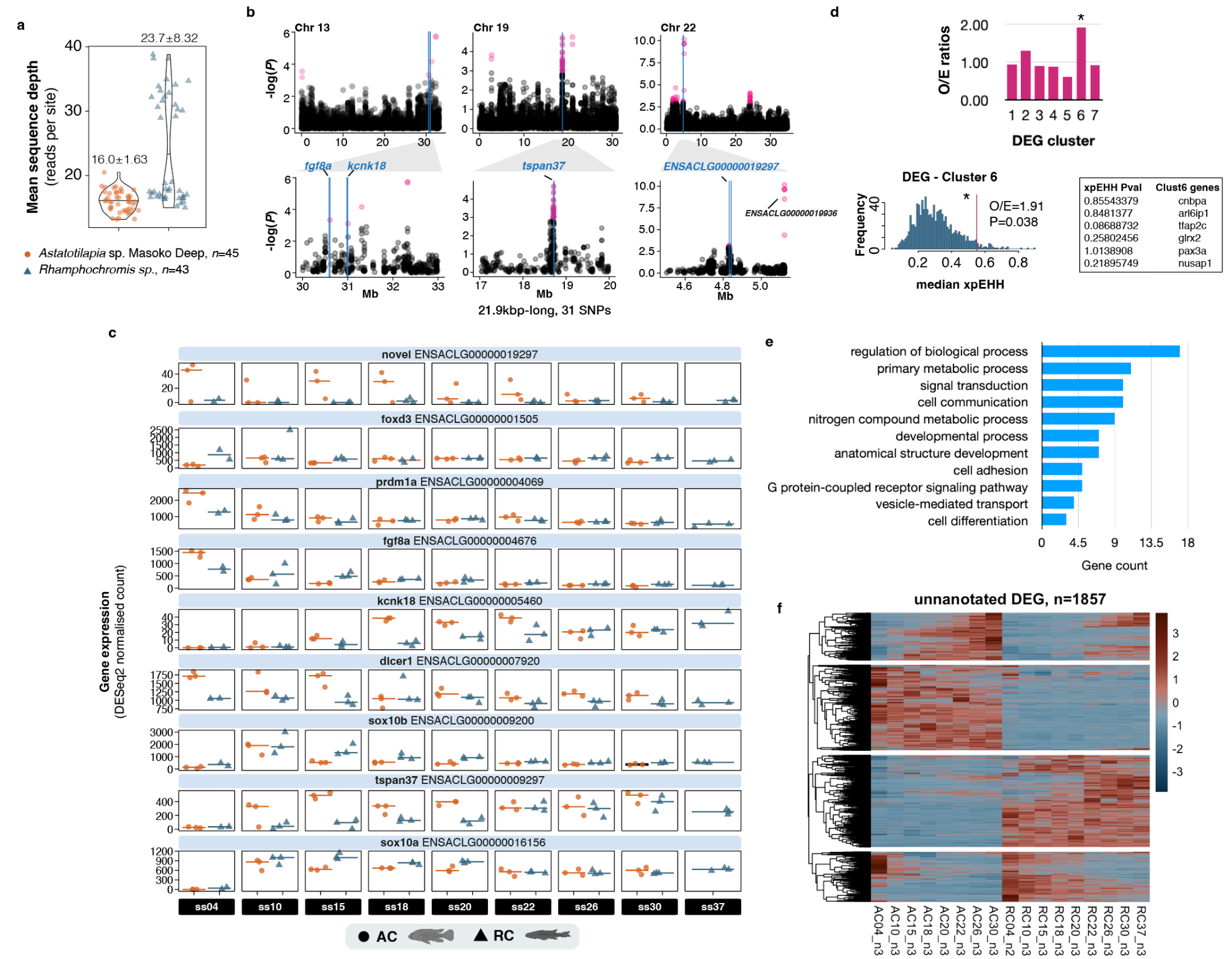
**

**Supplementary Figure S2. Comparative population genomics between AC and RC cichlid species identifies regions of positive selection associated with transcriptomic divergence. a**) Violin plot showing overall genome coverage for the two cichlid population used genomics analyses (horizontal bars for median values). **b)** Close-up genome-browser views focusing on the DEG candidates associated with xpEHH peaks shown in Fig. 2c. **c)** Plots of expression values for some DEG candidates associated with outlier regions of putative signatures of selection (refer to Fig. 2c); horizontal bars show median values. **d)** Upper panel: observed vs. expected ratios of xpEHH significance enrichment for all the NC-DE genes belonging to each DEG cluster. Expected values were computed through 1000 random iterations. All NC-DE genes from cluster 6 show significant enrichment for xpEHH scoresz, indicative of signature of positive selection. Lower panel: O/E distributions of xpEHH for NC-DEG belonging to cluster 6 (listed on the left). **e)** Top 10 GO categories of DEGs associated with xpEHH outlier peaks. **f)** Heatmap showing scaled gene expression for the 1857 DE unannotated genes.
