# Supplementary material for: Genetic and Developmental Divergence in the Neural Crest Program between Cichlid Fish Species
Source: Mol Biol Evol. 2024 Oct 16;41(11):msae217. doi: 10.1093/molbev/msae217 (PMC11558072; doi:10.1093/molbev/msae217)
Supplement: msae217_Supplementary_Data [file msae217_supplementary_data.zip › Supplementary Figure S3.docx]

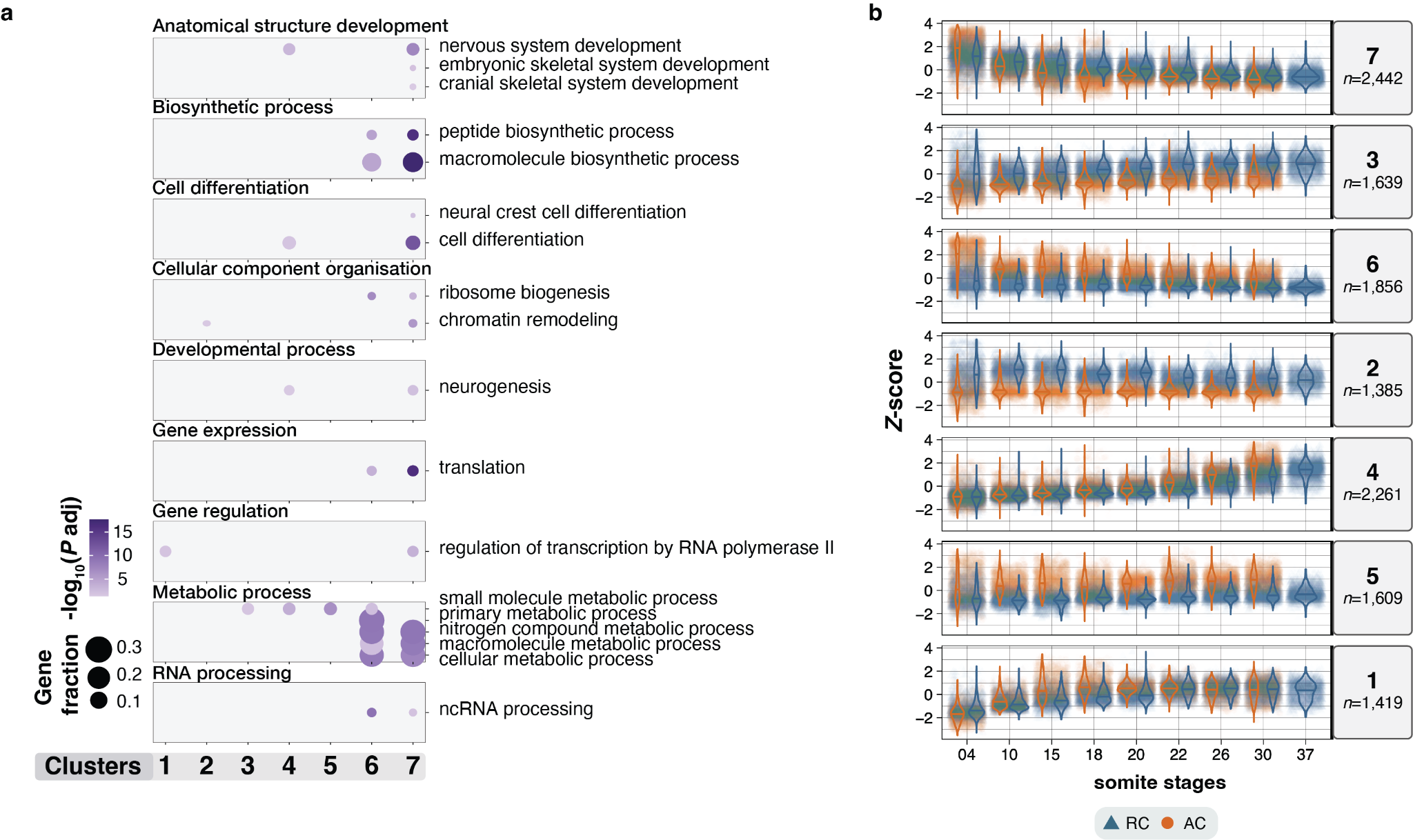


**Supplementary Figure S3. Unbiased, hierarchical clustering of DEGs based on gene expression patterns into seven distinct clusters.** **a)** Distinct Gene Ontology categories are significantly enriched in each of the seven clusters of gene expression identified**. b)** Gene expression dynamics (*Z*-score, scaled normalized gene count) for all DEGs across all somite stages according to seven gene expression clusters identified in Fig. 2a. Hpf, hour post-fertilisation. AC, *Astatotilapia calliptera* ‘Mbaka’; RC, *Rhamphochromis* sp. ‘chilingali’.
