# Supplementary material for: Genetic and Developmental Divergence in the Neural Crest Program between Cichlid Fish Species
Source: Mol Biol Evol. 2024 Oct 16;41(11):msae217. doi: 10.1093/molbev/msae217 (PMC11558072; doi:10.1093/molbev/msae217)
Supplement: msae217_Supplementary_Data [file msae217_supplementary_data.zip › Supplementary Figure S4.docx]

**
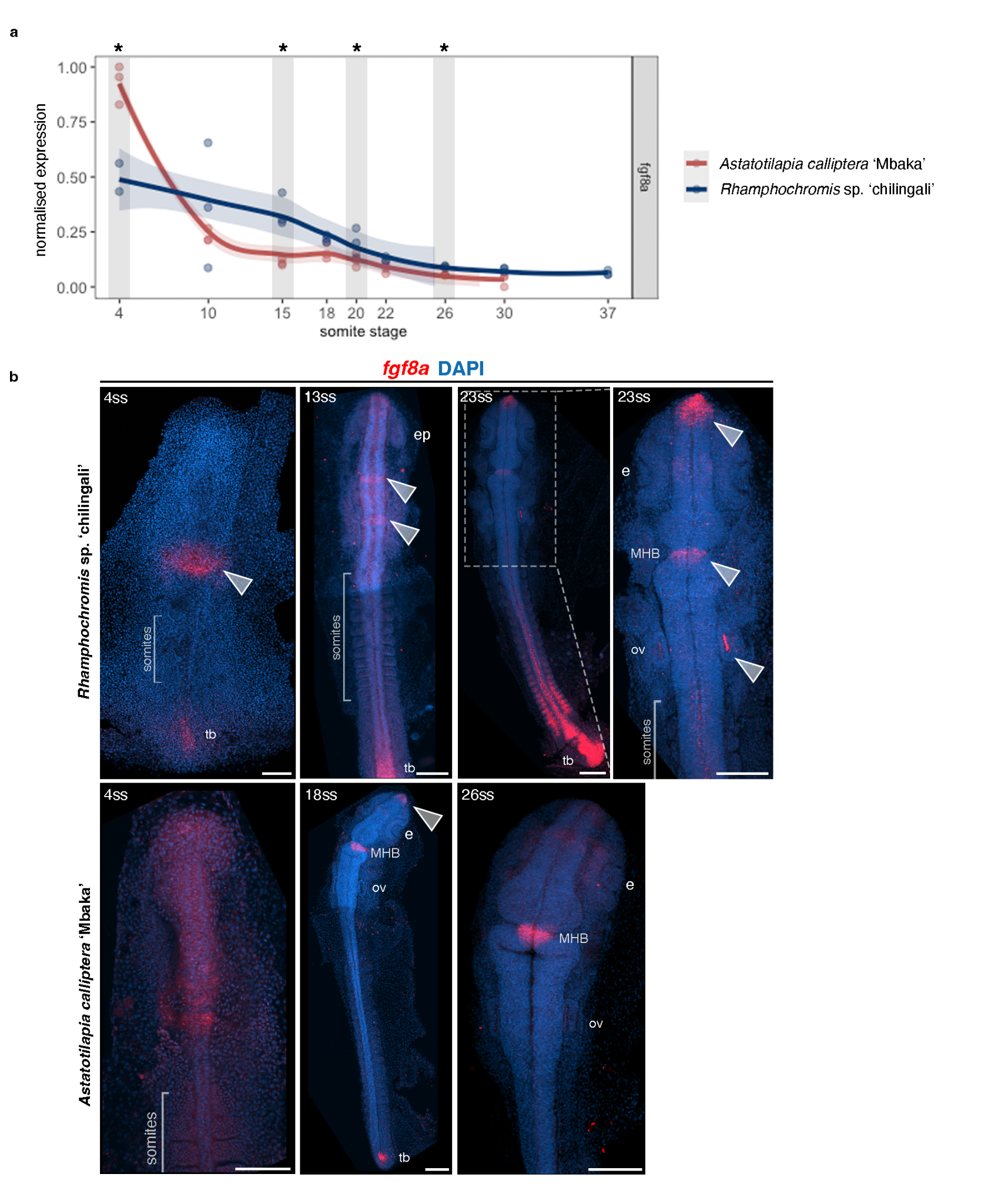
**

**Supplementary Figure S4. *fgf8a* expression patterns in cichlid embryos suggest a role in brain development during somitogenesis rather than the neural crest.** **a**) Significant differences in expression levels of *fgf8a* were observed at multiple stages of somitogenesis. Shaded panels and asterisks denote stages of differential expression between species with log_2_ fold change over 1.5 and p-adj < 0.05. **b**) Expression patterns of *fgf8a* in cichlids at selected stages of somitogenesis. Note the difference in expression domains between species at 4ss. At later stages, in addition to strong expression at the midbrain-hindbrain boundary (MHB) and anterior-most end tip of the forebrain, *fgf8a* transcripts were also detected in the tailbud (arrowheads). Expression in otic vesicles was only observed in *Rhamphochromis*. ep - eye primordium, e - eye, MHB - midbrain-hindbrain boundary, ov - otic vesicle, tb - tailbud. Scale bar = 100 μm.
