# Supplementary material for: Genetic and Developmental Divergence in the Neural Crest Program between Cichlid Fish Species
Source: Mol Biol Evol. 2024 Oct 16;41(11):msae217. doi: 10.1093/molbev/msae217 (PMC11558072; doi:10.1093/molbev/msae217)
Supplement: msae217_Supplementary_Data [file msae217_supplementary_data.zip › Supplementary Figure S5.docx]

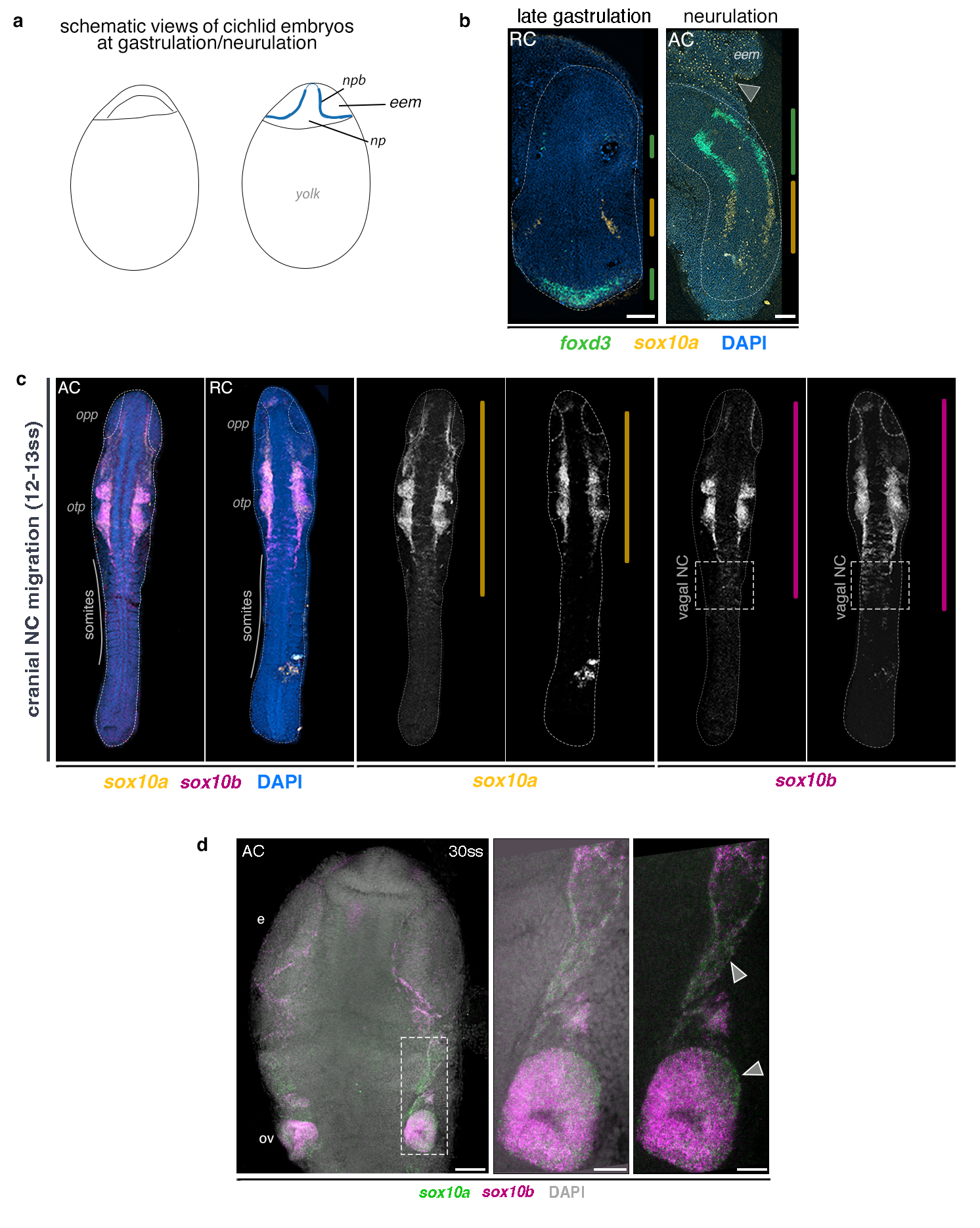


**Supplementary Figure S5. Temporal divergence between *sox10* paralogs and novel expression domain of *sox10a*. a-b)** In embryos undergoing gastrulation (a and b left) and neurulation (a and b right), *sox10a* expression was detected prior to *sox10* in domains distinct from *foxd3*, a marker of early neural crest cells. **c)** Migration of *sox10b+* vagal NC cells (axial level of somites 1-5) is ongoing in both species at 12ss, indicating synchronized progression of delamination and migration along the anterior-posterior axis. In contrast, cells expressing *sox10a* show variation between species. **d)** *sox10a* and *sox10b*, although largely co-expressed in neural crest cells during somitogenesis, were also detected in distinct nuclei in cranial neural crest, indicating paralog-specific cell populations (gray arrowheads). *sox10a* depicted in green to improve visual distinction of HCR signals. Note that, unlike zebrafish, epiboly in cichlids is not complete until late segmentation/early pharyngula stages (Marconi et al. 2023). AC - *Astatotilapia calliptera* ‘Mbaka’, eem - extra-embryonic membrane, np - neural plate, npb - neural plate border, RC - *Rhamphochromis* sp. ‘chilingali’, ss - somite stage. Scale bar = 100 μm, 50 μm in d left panel, and 25 μm in d central and left panel.
