# Supplementary material for: Genetic and Developmental Divergence in the Neural Crest Program between Cichlid Fish Species
Source: Mol Biol Evol. 2024 Oct 16;41(11):msae217. doi: 10.1093/molbev/msae217 (PMC11558072; doi:10.1093/molbev/msae217)
Supplement: msae217_Supplementary_Data [file msae217_supplementary_data.zip › Supplementary Figure S6.docx]

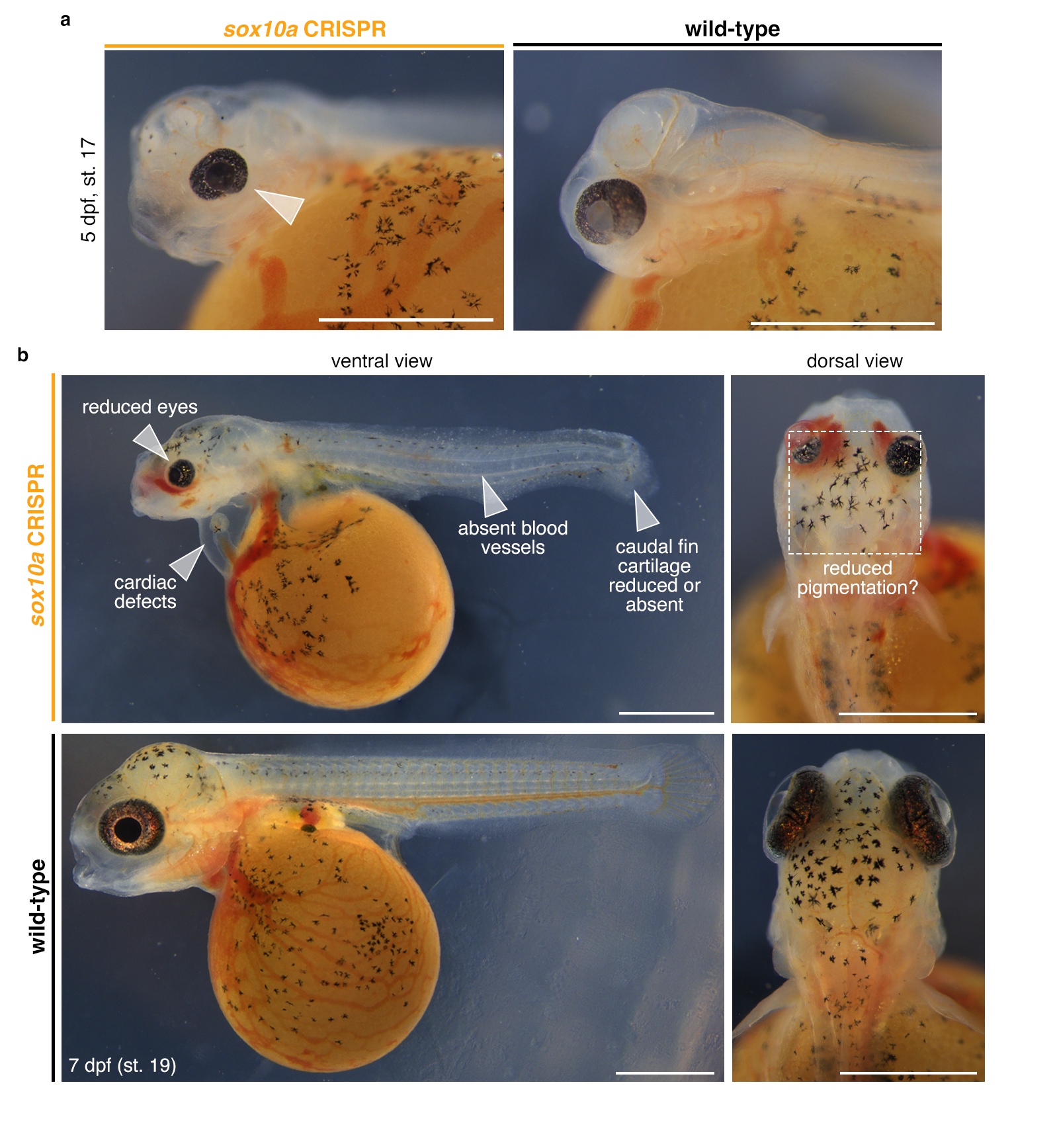


**Supplementary Figure S6. *sox10a* CRISPR embryos display multiple developmental defects. a)** Ocular defects, particularly reduced eye size (gray arrowhead), were observed in *sox10a* KO embryos by 5 dpf (st. 17). **b)** In addition to craniofacial cartilage and ocular malformations, embryos inspected at 7 dpf (st. 19) were also characterized by aberrations of cardiac and circulatory system as well as caudal fin cartilage development. The melanophore-based pigmentation of the dorsal cranium also appeared reduced compared to wild-type, however this could be explained by the reduced surface area of the pigmented region in mutants with severe craniofacial phenotypes. Dpf - days post-fertilization; st - stage. Scale bars = 1mm.
