# Supplementary material for: Genetic and Developmental Divergence in the Neural Crest Program between Cichlid Fish Species
Source: Mol Biol Evol. 2024 Oct 16;41(11):msae217. doi: 10.1093/molbev/msae217 (PMC11558072; doi:10.1093/molbev/msae217)
Supplement: msae217_Supplementary_Data [file msae217_supplementary_data.zip › Supplementary Figure S7.docx]

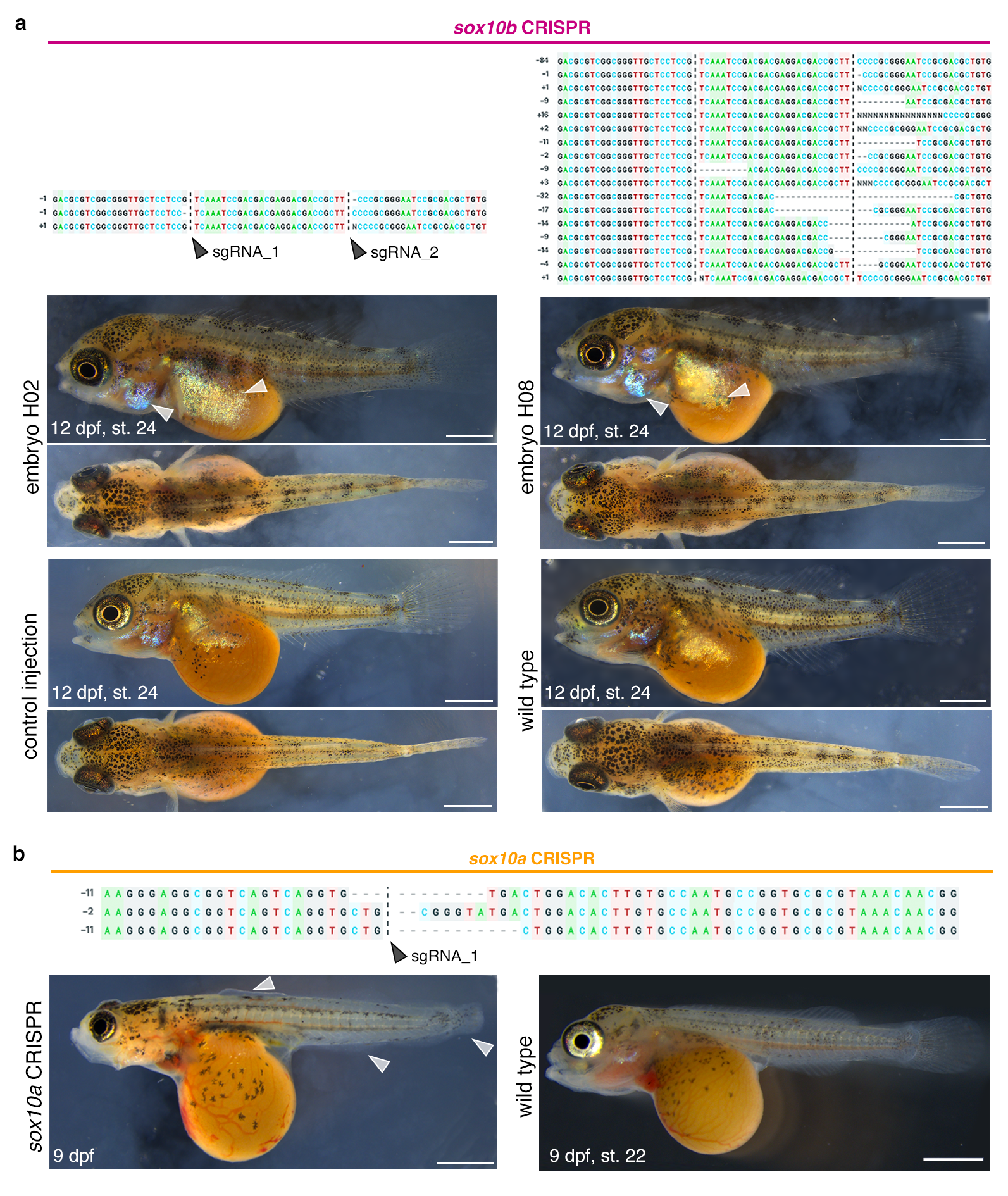


**Supplementary Figure S7.** **Examples of CRISPR/Cas9-induced mutant genotypes and associated phenotypes in *Astatotilapia calliptera* ‘Mbaka’.** Multiple sequence alignments of Sanger sequencing, showing deletions or insertions in exon 1 of *sox10b* (**a**) and *sox10a* (**b**), frequently resulting in frameshifts. **a)** *sox10b* CRISPR embryos at 12 dpf (st. 24, top row) have mild pigmentation abnormalities compared to control and wildtype clutch mates (bottom row), including increased iridophore coverage on the yolk and operculum (gray arrowheads). **b)** In addition to defects of the craniofacial cartilages **(Fig. 6),** surviving *sox10a* CRISPR fish have drastically reduced dorsal, anal and caudal fins (gray arrowheads), likely due to abnormal development of the cartilaginous fin rays.
