# Supplementary material for: Genetic and Developmental Divergence in the Neural Crest Program between Cichlid Fish Species
Source: Mol Biol Evol. 2024 Oct 16;41(11):msae217. doi: 10.1093/molbev/msae217 (PMC11558072; doi:10.1093/molbev/msae217)
Supplement: msae217_Supplementary_Data [file msae217_supplementary_data.zip › Supplementary_Table_10_Summary_mutant_phenotypes.pdf]

Supplementary Table 6. Summary of *sox9* and *sox10* paralog mutant phenotypes reported in published literature.

	sox9a	***sox9b***	***sox10a***	***sox10b***
Lake Malawi cichlids *Astatotilapia calliptera* and *Rhamphochromis* sp. 'chilingali' (this study)	/	/	Craniofacial cartilage, cardiac, ocular and pigmentation defects	Pigmentation defects
Nile tilapia (*Orechromis niloticus*)	Cartilage defects[1]	/	/	/
medaka (*Oryzias latipes*)	/	Pigmentation defects[2]	Pigmentation defects[3]	Pigmentation defects[3]
Zebrafish (*Danio rerio*)	Cartilage defects[4]	Cartilage defects[4], peripheral neuron and glial defects[5]	NA	Pigmentation defects[6,] peripheral neuron and glial defects[7-9]
	SOX9		***SOX10***	
Mouse (*Mus musculus*)	Skeletal malformations[10]		Aberrations of cranial nerve and ganglia morphology[11]	

/ - mutant phenotype unknown
NA – gene absent

1 - Li et al. 2023 *Zoological Research*
2 - Tsunogai et al. 2021 *Development, Growth & Differentiation*
3 - Nagao et al. 2018 *PLoS Genetics*
4 - Yan et al. 2005 *Development*
5 - Carney et al. 2006 *Development*
6 - Kelsh 2000 *BioEssays*
7 - Kelsh and Eisen 2000 *Development*
8 - Elworthy et al. 2000 *Mechanisms of Development*
9 - Delfino-Machin et al. 2017 *PLoS ONE*
10 - Wagner et al. 1994 *Cell*
11 - Kuhlbrodt et al. 1998 *Journal of Neuroscience*
